# CyTargetLinker: A Cytoscape App to Integrate Regulatory Interactions in Network Analysis

**DOI:** 10.1371/journal.pone.0082160

**Published:** 2013-12-05

**Authors:** Martina Kutmon, Thomas Kelder, Pooja Mandaviya, Chris T. A. Evelo, Susan L. Coort

**Affiliations:** 1 Department of Bioinformatics - BiGCaT, NUTRIM School for Nutrition, Toxicology and Metabolism, University of Maastricht, Maastricht, The Netherlands; 2 Netherlands Consortium for Systems Biology (NCSB), Amsterdam, The Netherlands; 3 TNO, Research Group Microbiology and Systems Biology, Zeist, The Netherlands; University of Erlangen-Nuremberg, Germany

## Abstract

**Introduction:**

The high complexity and dynamic nature of the regulation of gene expression, protein synthesis, and protein activity pose a challenge to fully understand the cellular machinery. By deciphering the role of important players, including transcription factors, microRNAs, or small molecules, a better understanding of key regulatory processes can be obtained. Various databases contain information on the interactions of regulators with their targets for different organisms, data recently being extended with the results of the ENCODE (Encyclopedia of DNA Elements) project. A systems biology approach integrating our understanding on different regulators is essential in interpreting the regulation of molecular biological processes.

**Implementation:**

We developed CyTargetLinker (http://projects.bigcat.unimaas.nl/cytargetlinker), a Cytoscape app, for integrating regulatory interactions in network analysis. Recently we released CyTargetLinker as one of the first apps for Cytoscape 3. It provides a user-friendly and flexible interface to extend biological networks with regulatory interactions, such as microRNA-target, transcription factor-target and/or drug-target. Importantly, CyTargetLinker employs identifier mapping to combine various interaction data resources that use different types of identifiers.

**Results:**

Three case studies demonstrate the strength and broad applicability of CyTargetLinker, (i) extending a mouse molecular interaction network, containing genes linked to *diabetes mellitus*, with validated and predicted microRNAs, (ii) enriching a molecular interaction network, containing DNA repair genes, with ENCODE transcription factor and (iii) building a regulatory meta-network in which a biological process is extended with information on transcription factor, microRNA and drug regulation.

**Conclusions:**

CyTargetLinker provides a simple and extensible framework for biologists and bioinformaticians to integrate different regulatory interactions into their network analysis approaches. Visualization options enable biological interpretation of complex regulatory networks in a graphical way. Importantly the incorporation of our tool into the Cytoscape framework allows the application of CyTargetLinker in combination with a wide variety of other apps for state-of-the-art network analysis.

## Introduction

Completion of the human genome project in 2003 generated a wealth of information about the human genetic code [1]. Approximately 25,000 gene coding regions were defined. However, understanding of the regulation of gene expression, protein synthesis and activity is far from complete. Recently, the ENCODE project, whose main goal was to identify all the functional elements in the human genome sequence, revealed novel insights in genetic regulation [2], [3]. It still remains a challenge to combine the new insights with existing knowledge and to understand the regulation of biological processes in detail. Many known biological processes are represented in various online repositories, like WikiPathways [4] and Reactome [5]. These processes contain genes, proteins and/or metabolites, their molecular interactions and reactions, but little regulatory information is present.

Regulation of gene expression, protein synthesis and activity occurs at different levels. Whereas gene expression is influenced by epigenetic factors and/or transcription factor (TF) binding, protein synthesis can be regulated by microRNAs (miRNAs). TFs are proteins that bind to a specific DNA sequence, i.e., the transcription factor binding site (TFBS). They either activate or repress transcription. miRNAs are small, non-coding RNA molecules that bind to miRNA-target regions in the mRNA. Upon binding miRNAs either repress translation or cleave the mRNA sequence. They are able to influence the synthesis of many proteins or even those involved in entire pathways, making them important molecules in harmonised regulation. Another group of regulatory effects are post-translational modifications which can influence protein activity. These modifications include phosphorylation, acetylation, palmitoylation and many more. In addition, small molecules such as metabolites or drugs can play a role as regulators in cellular pathways.

A systems biology approach in which interactions from different resources are combined, visualised and analysed together is an intuitive way to decipher complex biological processes. A commonly used framework to visualise and analyse biological networks is Cytoscape [6]. Its modular structure and possibilities to extend with additional functionalities through apps (formerly known as plugins) is discussed in “A travel guide to Cytoscape plugins” [7]. At the moment a few Cytoscape apps are available that either extend networks with other types of molecular interaction data or focus on one specific type of regulatory interaction. However, a user-friendly tool to combine and integrate various types of regulation is still needed. In this paper, we present a new Cytoscape app, CyTargetLinker, to automatically add regulatory interactions to biological networks to allow their inclusion in the network analysis process. CyTargetLinker is not restricted to one specific organism or regulatory interaction type and it leaves the selection of relevant and/or preferred interaction databases entirely to the user. The incorporation of our tool into the Cytoscape framework allows its application in combination with several community-contributed apps for data visualization and advanced network analysis.

### Implementation

CyTargetLinker is an open source app developed for the network visualization and analysis tool Cytoscape [6] and can be installed through the app manager in Cytoscape 2.8 or 3.x. The source code is available on Github (https://github.com/mkutmon/cytargetlinker).

CyTargetLinker allows users to build regulatory networks to obtain a more complete view of biological systems. The regulatory interactions used in CyTargetLinker are derived from so called *regulatory interaction networks* (RegINs) that are either provided on the CyTargetLinker website or can be created by the user. The creation, application and content of RegINs is explained below. All functionalities of CyTargetLinker are described in the “CyTargetLinker workflow” section.

### Regulatory interaction networks

A RegIN is a network containing regulatory interactions that are often derived from online interaction databases. The networks are stored in XGMML (the eXtensible Graph Markup and Modelling Language) format, which is supported by Cytoscape. Each regulatory interaction consists of two nodes, a source (regulatory component) and target biomolecule, connected through one directed edge. A collection of RegINs for different species and interaction types is provided on the CyTargetLinker website (http://projects.bigcat.unimaas.nl/cytargetlinker/regins), and is described in more details in Table 1. In addition, we provide documentation on how to create your own RegIN (see supporting information, File S1). The app is not restricted to the RegINs provided and the user can choose which interaction types and databases should be used in the integration process.

**Table 1:** Regulatory interaction network files.

Database	version	Type	Human	Mouse	Rat	Zebrafish
MicroCosm	version 5	predicted MTI	541,039	494,822	511,057	121,992
TargetScan	release 6.2	predicted MTI	511,040	186,431	-	-
miRTarBase	release 3.5	validated MTI	3,597	712	278	104
miRecords*	version 4	validated MTI	1,752	395	161	48
ENCODE	2012	proximal TF-target	24,111	-	-	-
ENCODE	2012	distal TF-target	18,240	-	-	-
TFe	2012-10-12	TF-target	1,531	847	-	-
DrugBank	version 3	drug-target	14,070	-	-	-

Subset of the RegINs (regulatory interaction networks) available for download on the CyTargetLinker website. All RegIN networks support the following identifier systems: (i) for genes/proteins 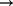 Ensembl, NCBI gene, UniProt, (ii) for miRNAs 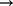 miRBase accession number and ids, and (iii) for drugs 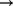 DrugBank. (* Redistribution of data not allowed, but RegIN can be created with our provided conversion script).

A set of RegINs can be seen as a collection of online interaction databases that are formatted in the same way so they can be combined in the integration and analysis process. For the available RegINs, in order to be able to jointly use them, one unifying identifier system was used: the Ensembl gene identifier [8] was chosen for genes, the miRBase [9] accession number for miRNAs and DrugBank [10] identifiers for drugs. The identifier mapping was performed using the BridgeDb mapping framework [11]. In addition to the main identifier system, the RegINs contain additional systems (e.g. NCBI gene [12] and UniProt [13] for genes/proteins) to give the user more freedom to choose the identifier system in the initial network which has to match to that used in the RegIN. In case the identifier system is not supported by the RegINs to be used, the user can use the CyThesaurus [11] app in Cytoscape to map the identifiers to one of the supported systems.

### CyTargetLinker workflow

**Figure 1:**
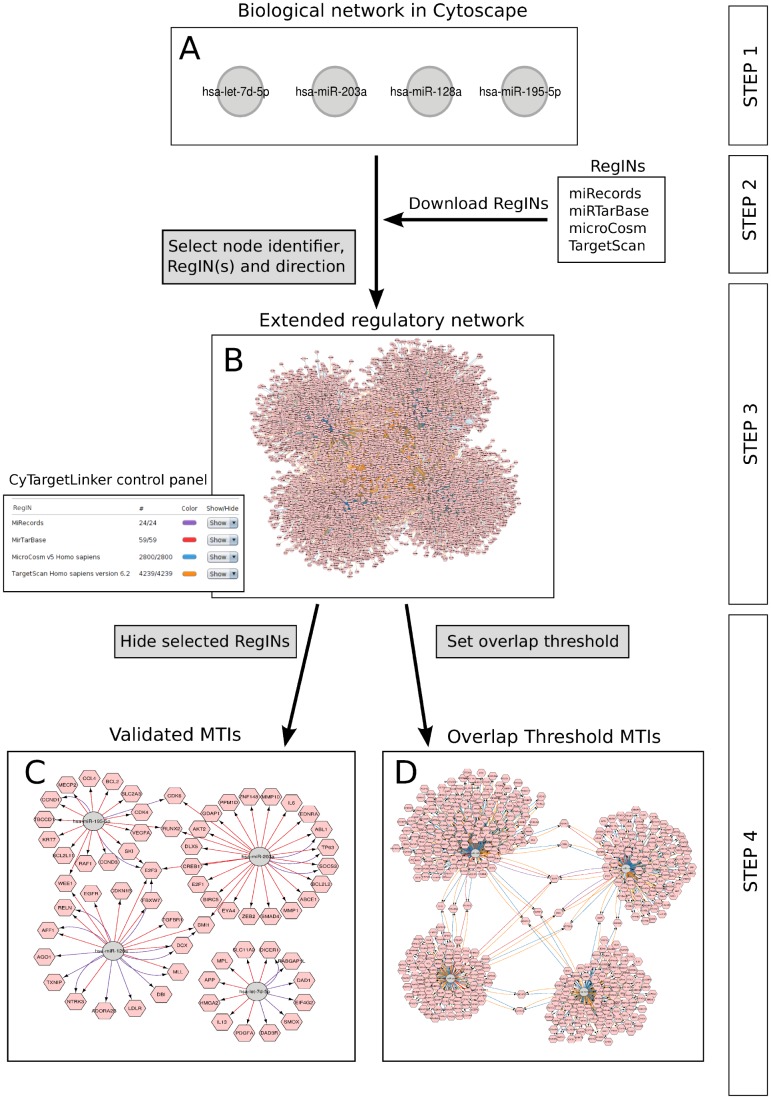
CyTargetLinker workflow. **Step 1:** Four miRNAs known to be involved in prostate cancer [27] are visualised in a Cytoscape network (A). The miRNAs are annotated with miRBase accession numbers and ids. **Step 2:** The regulatory interaction networks (RegINs) harbouring miRNA-target interactions (MTIs), either validated (miRecords and miRTarBase) or predicted (microCosm and TargetScan), are downloaded from http://projects.bigcat.unimaas.nl/cytargetlinker/regins. **Step 3:** Known targets are integrated (B) after specifying the miRBase accession number column, the RegINs directory and the direction “*Add Targets*” in the CyTargetLinker dialog. In the resulting network miRNAs and target genes are defined as grey circles and pink hexagons, respectively. The predicted MTIs are visualised in orange (TargetScan: 4239) and blue (microCosm: 2800) and the validated MTIs in red (miRTarBase: 59) and purple (miRecords: 24), as shown in the control panel. **Step 4:** The hide/show and overlap threshold functions were used to visualise validated interactions exclusively or to show the overlap in MTI coverage. In the validated network only the MTIs in miRecords and miRTarBase are visualised by hiding MTIs in TargetScan and microCosm (C). In the overlap network the MTIs present in two or more RegINs are shown by setting the threshold to 2 (D).

CyTargetLinker enables the enrichment of biological networks with regulatory information in a user-friendly and flexible manner. The CyTargetLinker workflow will now be discussed in detail and is illustrated by an example in Figure 1.

The first step is to load or create a biological network in Cytoscape. Starting from a protein-protein network, a biological pathway or unconnected gene nodes, the initial network that will be extended with regulatory information can be very different. In each case the elements in the network should be annotated using one of the supported identifier systems. The second step is to download or create RegINs, as described in the next section. In the third step the CyTargetLinker integration process is started in Cytoscape. In the dialog the user selects the biological network, the node identifier attribute and the local directory containing the downloaded RegIN files. Thereafter, the direction of the interaction should be selected. It is possible to only add targets, regulators or both (default). CyTargetLinker will extract only those regulatory interactions from the provided RegINs, in which one of the nodes in the initial network is a participant, either regulator or target. This reduces the amount of memory needed, speeds up the integration process, and makes CyTargetLinker scalable to large regulatory networks.

After the extension of the network the initial network nodes are visualised as grey circles whereas the added nodes are shown as pink hexagons (see Figure 1B). Moreover, the edge colour defines in which RegIN an interaction is present. If an interaction is supported by more than one RegIN, CyTargetLinker will add one differently coloured edge for each RegIN. In the accompanying control panel the interaction colour can be changed and the number of added interactions per RegIN is listed. In the fourth step the visualization of the regulatory network can be adapted by using the *hide/show* and/or *overlap threshold* function. The *hide/show* functionality enables the temporary removal of specific RegINs and thereby showing only the interactions from a subset of the loaded RegINs (see Figure 1C). The *overlap threshold* functionality makes it possible to show only the interactions that are supported by a defined number of RegINs or more (see Figure 1D). Both functions can be applied and restored in the same network window.

## Results

The strength and broad applicability of CyTargetLinker will be demonstrated by three different case studies. In these studies the currently available RegINs will be used for extending biological networks. In Table 1, a subset of the downloadable RegINs present at the CyTargetLinker website is shown. The RegINs are generated from the latest database version and will be updated once a new version is available and accessible. The older versions will stay available in an archive.

### Use cases

**Case study 1 - Enrichment of a mouse molecular interaction network, containing genes linked to diabetes mellitus, with miRNA information.** The first case study demonstrates that CyTargetLinker is not limited to human networks, but can be used for other species as well. The threshold functionality is applied to show only interactions that are supported by at least two miRNA-target interaction (MTI) databases.

*Diabetes mellitus* is a group of metabolic diseases. The two major types are type 1 and type 2 which are characterised by impaired insulin production or insulin resistance, respectively. Worldwide the prevalence of *type 2 diabetes mellitus* (T2DM) is increasing dramatically. Although a strong environmental component is present, there is compelling evidence that genetic factors are involved in the pathogenesis of T2DM [14]. It is important to decipher the genes involved and to understand their regulation. Diabetic mouse models are often used to measure gene expression on a large scale in tissues like adipose, skeletal muscle and liver. The genes linked to *diabetes mellitus* can be functionally annotated using the terms in the disease category of MeSH (Medical Subject Headings) [15]. CyTargetLinker can be used to examine the possible role of miRNA regulation of genes known to be linked to *diabetes mellitus*.

**Figure 2:**
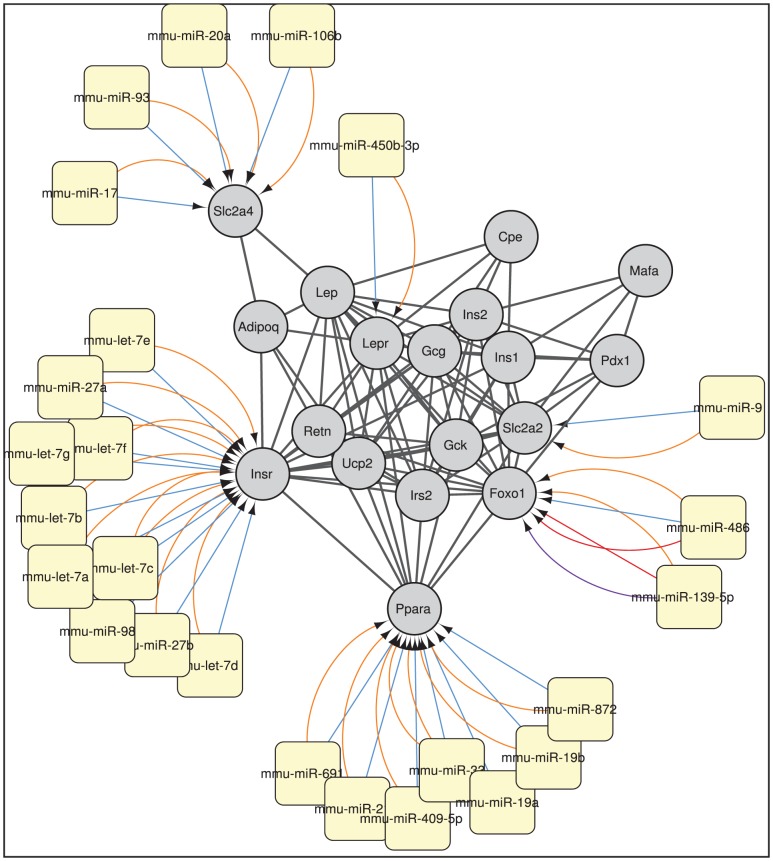
miRNA regulation of genes associated with *diabetes mellitus*. Genes and miRNAs are visualised as grey circles and yellow rounded rectangles, respectively. The names of the genes and miRNAs are displayed on the nodes. The genes linked to the MeSH term *diabetes mellitus* were obtained using Gene2MeSH (http://gene2mesh.ncibi.org/). The molecular interactions between the genes were obtained from the STRING database and are visualised in black. The MTIs originate from microCosm (486), TargetScan (207), miRTarBase (5) and miRecords (2), and are coloured in blue, orange, red and purple, respectively.The overlap threshold function was applied to show only MTIs present in at least two RegINs.

A mouse molecular interaction network of genes linked to the MeSH term *diabetes mellitus* was obtained from Gene2MeSH [16] and the STRING database [17]. In total 18 proteins are associated with *diabetes mellitus* and are known to interact with each other (see supporting information, File S2). To get a better insight in how the genes are regulated by miRNAs, validated and predicted miRNAs were added and the overlap of the MTIs present in two or more databases was selected with CyTargetLinker (see Figure 2). After applying overlap threshold, 50 MTIs remain in the extended network originating from miRecords (1), miRTarBase (2), microCosm (24) and TargetScan (25). In the extended molecular interaction network only 6 out of 18 genes interact with mostly predicted miRNAs that were present in 2 or more interaction databases. Whereas LEPR (leptin receptor) and SLC2A2 (GLUT2) interact with only one miRNA, INSR (insulin receptor) and PPARa (peroxisomal proliferator activated receptor alpha) are highly regulated by 7 and 10 miRNAs, respectively. It is well known that the activation of the nuclear receptor, PPARalpha, has beneficial effects in T2DM. PPAR agonists are used as antidiabetic drugs to treat the symptoms of T2DM. Identifying which miRNAs interact with PPARalpha could lead to novel pharmacological targets. In this use case CyTargetLinker can be used to either identify miRNAs of interest or to confirm recent findings. For example, the regulation of PPARalpha by miR-21, present in the extended network, was published in 2011 in a liver study [18]. The other highly regulated gene, the insulin receptor, plays a key role in the insulin signalling pathway. Most miRNAs interacting with the insulin receptor in the extended network belong to the let-7 miRNA family (see Figure 2). Interestingly, Forst and Olson [19] showed that the let-7 family controls glucose homeostasis and insulin sensitivity in mice. Their study confirms that in liver and muscle the let-7 family regulates the insulin receptor as shown in the extended network.

**Case study 2 - Extension with ENCODE TF regulation information of a molecular interaction network of human DNA repair genes and its analysis.** The second case study demonstrates how CyTargetLinker can be applied in combination with other Cytoscape apps. Moreover, it shows that published regulatory interaction data can be easily converted into a RegIN and implemented into the CyTargetLinker workflow. By using the available core functionalities of Cytoscape is it possible to adjust the colour and size of a node and to perform commonly used network analyses in the extended network.

The ENCODE project aims to delineate all functional elements encoded in the human genome [2]. Since 2003 it has generated a wealth of information on regulatory elements. Gerstein and colleagues used the recently published TF binding data to analyse differential patterns in promoter proximal and distal regulatory regions [3]. TFs bind to specific sites, transcription factor binding sites (TFBS), that can be proximal or distal to a transcription start site [20]. Distinguishing these two types of TF-regulation gives a more distinctive view on how gene expression can be influenced.

**Figure 3:**
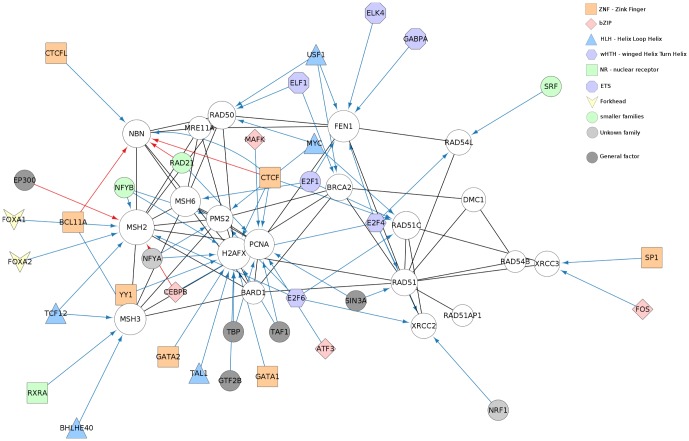
Extend a GeneMania network with TF data from the ENCODE project. A set of known DNA repair genes (grey circled nodes) were used as input for the GeneMania app in Cytoscape. Physical interactions (black) between the query genes were added by GeneMania. Every node in a GeneMania network has an attribute “Entrez gene identifier” which was used by the CyTargetLinker app to extend the network with TF-gene interactions from the ENCODE networks. The integration direction was selected as “*Add regulators*”, indicating that the app should look for interactions that target the input genes. The colours and shapes of the TFs are based on the provided TF family information in the ENCODE project [3]. The ENCODE project studied proximal and distal TF regulation which are indicated in this figure as blue and red edges.

We used the proximal and distal regulatory interaction data provided by Gerstein and generated RegINs for both. A set of known DNA repair genes were used as input for the GeneMANIA app [21] in Cytoscape to create a molecular interaction network (see supporting information, File S3). GeneMANIA identifies the most related genes to a query gene set using a guilt-by-association approach. With CyTargetLinker the DNA repair network was enriched with the two types of regulatory data. Next, we used Cytoscape's VizMapper to shape and colour the TF nodes according to their TF family. To identify the genes highly regulated by TFs, the indegree was calculated and represented as the size of the gene nodes, see Figure 3. From the network it is immediately clear that the H2AFX (H2A histone family, member X) gene is highly regulated by proximal TFs. Moreover, the NBN and MSH2 genes are regulated by both proximal and distal TFs.

**Case study 3 - Enrichment of a human pathway from WikiPathways with miRNAs, TFs and drugs targeting the genes and gene products in the pathway.** Case study 3 highlights the power of CyTargetLinker to build an extensive regulatory interaction network integrating a wide range of known interactions. This network can be used as a starting point for various network analysis approaches to filter out regulatory interactions that are relevant in a given context. The integration of different regulatory elements together allows the researcher to get a more complete view of possible regulatory mechanisms happening in a biological process.

**Figure 4:**
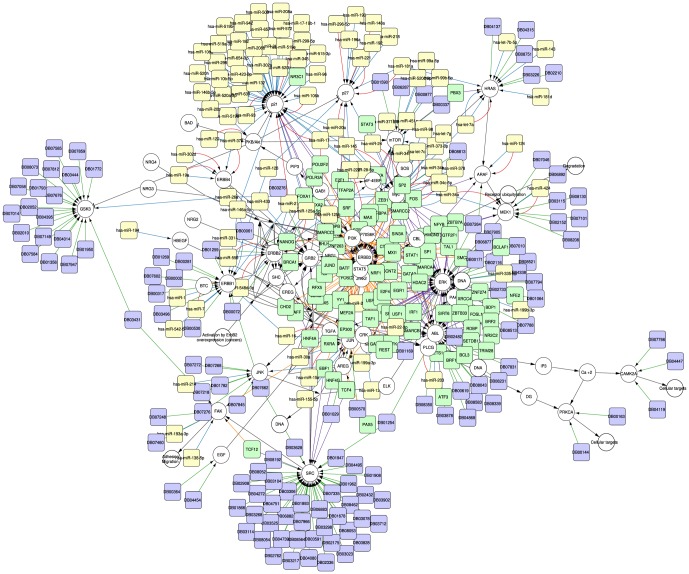
Meta network of the ErbB signaling pathway containing miRNA, TF and drug regulation. The ErbB signaling pathway (http://wikipathways.org/index.php/Pathway:WP673) from WikiPathways was extended with TFs, miRNAs and drugs regulating the elements in the pathway. The nodes from the original pathway are coloured in grey, TFs in green, miRNAs in yellow and drugs in purple. 258 TF-gene interactions from ENCODE, 162 miRNA-target interactions from the two validated miRNA-target RegINs (miRTarBase and miRecords) and 138 drug-target interactions from DrugBank were added to the network. The edges are coloured based on the RegIN source, purple and orange for ENCODE (proximal and distal), blue and red for miRTarBase and miRecords, and green for DrugBank.

The initial network represents the ErbB signaling pathway which was loaded through the WikiPathways web service provided by the GPML app [22] (see supporting information, File S4). Insufficient ErbB signaling may cause the development of neurodegenerative diseases, such as multiple sclerosis and Alzheimer's disease. Furthermore, excessive ErbB signaling is associated with the development of various types of solid tumours [23]. The pathway contains 69 genes, proteins and metabolites (plus 13 group nodes representing grouped genes or proteins in the original pathway diagram) and 93 edges. The network was extended with three different types of regulatory interactions, (i) drug-target interactions from DrugBank, (ii) proximal and distal TF-gene interactions from ENCODE and (iii) validated miRNA-target interactions from miRTarBase and miRecords, see Figure 4. In total, 558 regulatory interactions were integrated in the network, including 138 drug-target, 136 proximal TF-gene, 122 distal TF-gene and 162 validated miRNA-target interactions.

There are a few nodes that are mostly targeted by drugs, e.g. GSK3 or SRC and other nodes that are targeted by all types of regulators, e.g. ERK or HRAS. In terms of transcription factor regulation, some nodes are regulated in a proximal setting, e.g. ERK or ABL and others mostly in a distal way, e.g. ERBB3 or STAT5. Some TFs regulate different genes in different ways, e.g. TCF12 regulates FAK distal and SRC proximal. Since a few TFs were already present in the initial network, typical network motifs can be found, e.g. four feed forward loops regulating ERBB3 over Myc by CTCF, SMC3, RAD21 and GATA2. In this integration process the direction was set to include only regulators, so the target genes of MYC and JUN, two major TFs that are present in the pathway, are not added.

Related to miRNAs our analysis shows that p21 is mostly regulated by miRNAs and all the interactions are experimentally validated (references provided in edge attributes). Furthermore with the *overlap threshold* functionality, it can be visualised that only 30 out of 130 miRNA-target interactions are present in both miRTarbase and miRecords.

## Discussion

CyTargetLinker enables quick and extensive enrichment of biological networks with regulatory information. It encapsulates all the manual integration steps which include several tasks that require advanced programming knowledge. Therefore it can be used by researchers from different fields with or without prior knowledge in programming and network analysis. CyTargetLinker is open source and freely available through the app manager for Cytoscape 2.8 and 3.x. As mentioned in the introduction, there are a few other Cytoscape apps that provide partial similar functionality. miRScape, CluePedia [24], ConReg, and BioNetBuilder [25] are most related to CyTargetLinker, see Table 2.

**Table 2:** Comparison of available Cytoscape apps.

App name	Cytoscape version	Availability	Source	Data	Organisms
miRScape	2.6	only by contacting developer	Not available	data from miRo' knowledge base [28] (last updated in 2009)	Human
CluePedia	3.0	app manager but can only be used with a license key	Not available	microCosm (miRanda) and miRecords	Human
ConReg	2.8	plugin manager	Not available	databases, text-mining and TFBS predictions	8 model organisms
BioNetBuilder	2.6	plugin manager	Available with read access	DIP, BIND, Prolinks, KEGG, HPRD, BioGrid, GO	> 1000 organisms
CyTargetLinker	3.0	app manager	Open Source	not restricted, user can provide RegINs	not restricted

Overview of four Cytoscape apps that are most related to CyTargetLinker. This table provides a comparison of the availability and data used in the apps.

miRScape identifies function, disease or process associations between genes by using miRNA-target information. CluePedia integrates experimental data to identify gene interrelations revealed by correlation weights, miRNAs regulatory aspects, protein-protein interactions as well as the functional context, in conjunction with ClueGO [26]. ConReg visualises TF-target gene networks with data from regulatory databases, text-mining approaches and TFBS predictions. It stores regulatory relations for 8 model organisms and investigates their level of conservation in related species. Lastly, BioNetBuilder focusses on the creation of biological networks using interaction data from DIP, BIND, Prolinks, KEGG, HPRD, BioGrid and GO.

BioNetBuilder and miRScape have not been maintained since version Cytoscape 2.6 (released in 2010). While CluePedia is also available as a Cytoscape 3.x app, it requires a license key. CyTargetLinker is generic and does not focus on one specific regulatory interaction type like miRScape and CluePedia. ConReg focuses only on TF-gene interactions, especially the conversion of regulatory relations in other eukaryotic model organisms and it produces a predicted conserved network. BioNetBuilder and CyTargetLinker can be used for several different species, however BioNetBuilder does not include regulatory interactions. While BioNetBuilder focusses on the creation of biological networks, CyTargetLinker extends biological networks with regulatory information. One of the advantages of the CyTargetLinker app is that it is easily expandable, a new RegIN can be added at any time and it is even possible to include a new interaction type without updating the app. Thereby, the user can use self-created RegINs in addition to the ones we provide and he can select the set of RegINs that are most suitable for his research focus. CyTargetLinker is an open source project which allows the contribution and input of other scientists to better tackle their research questions.

## Conclusion

CyTargetLinker, our new Cytoscape app, enables scientists to integrate regulatory interactions into biological networks in a user-friendly and flexible manner. Various interactions, such as miRNA-target, TF-gene or drug-target, can be added, by themselves or combined. CyTargetLinker is not restricted to any organism and the commonly used identifiers for genes, proteins, and miRNAs are supported. The graphical representation in Cytoscape facilitates the identification of important regulatory interactions and can lead to new research hypotheses. The integration of CyTargetLinker into Cytoscape enables advanced network analysis and data visualization using functionality from other apps. This helps researchers to get a better understanding of the regulation of biological processes.

## Future Developments

Future work, by us or other contributing groups, will include the development of new app features and conversion scripts for more publicly available databases, as well as allowing the connection to online graph databases (e.g. Neo4j) and RDF triple stores directly. This would even further simplify the integration process because the user does not need to download the RegINs beforehand.

## Supporting Information

File S1 **Detailed description of the structure, content and creation of regulatory interaction networks (RegINs).**
(PDF)

File S2 **Supporting information for case study 1 which is described in the results section.** Description of the initial network of the use case and the overlap between the different miRNA-target databases for this use case.
(PDF)

File S3 **Supporting information for case study 2 which is described in the results section.** Description of the initial network of the use case and more detailed information about the transcription factors, e.g. transcription factor families.
(PDF)

File S4 **Supporting information for case study 3 which is described in the results section.** Contains the pathway diagram of the ErbB signaling pathway from WikiPathways.
(PDF)
