# Supplementary material for: CyTargetLinker: A Cytoscape App to Integrate Regulatory Interactions in Network Analysis
Source: PLoS One. 2013 Dec 5;8(12):e82160. doi: 10.1371/journal.pone.0082160 (PMC3855388; doi:10.1371/journal.pone.0082160)
Supplement: File S1 — Detailed description of the structure, content and creation of regulatory interaction networks (RegINs). (PDF) [file pone.0082160.s001.pdf]

Supporting Information: S1

Regulatory Interaction Networks (RegINs).

The regulatory interactions networks used by CyTargetLinker are stored in XGMML (the eXtensible Graph Markup and Modelling Language) format . Cytoscape provides a description of the format on http://wiki.cytoscape.org/XGMML.

RegINs provided on the CyTargetLinker website:

- Homo sapiens
  - miRTarBase 3.5
  - TargetScan 6.2
  - microCosm 5
  - ENCODE
  - TFe
  - DrugBank 3
- Mus musculus
  - miRTarBase 3.5
  - TargetScan 6.2
  - microCosm 5
- Rattus norvegicus
  - miRTarBase 3.5
  - microCosm 5
- Caenorhabditis elegans
  - miRTarBase 3.5
  - microCosm 5
- Danio rerio
  - miRTarBase 3.5
  - microCosm 5

All the conversion scripts are available on github: https://github.com/mkutmon/rin-creation

Users can always download a specific database version and build the RegIN XGMML file. We also provide the scripts for databases with a restrictive license. Since we are not allowed to redistribute the data, the users have to build the xgmml files manually. This process is described here: https://github.com/mkutmon/rin-creation/wiki/Create-miRecords-RegIN-files.

Users can request for additional databases via the contact form on the CyTargetLinker website: http://projects.bigcat.unimaas.nl/cytargetlinker/contact/. We are always looking for new regulatory interaction databases to extend our RegIN collection.

Creating your own RegIN:

A RegIN for CyTargetLinker needs a set of attributes for the graph, the node and edge elements:

Graph:

Attribute	*Description*
label	Name of the RegIN – will be displayed in the CyTargetLinker panel

Node:

Attribute	*Description*
identifiers	List of identifiers representing the node. Enables identifier resolution by providing several identifiers for one node, e.g Ensembl, NCBI Gene and/or UniProt for a gene product (see example below).
biologicalType (optional)	Attribute to identify the biological type of a node (if given used for node fill color), e.g. gene, miRNA, drug, transcription factor.
label (optional)	Name of the node that will be displayed on the network.

Edge:

Attribute	*Description*
datasource	Name of RegIN to identify source of interaction

The listed attributes, both required and option, will be used by CyTargetLinker. All other attributes provided in the RegIN will be loaded into the Cytoscape table and are visible to the user as well.

Excerpt of a RegIN containing two nodes and one edge:

hsa-miR-449a **- *MTI* ->** HES1


```xml
<?xml version="1.0" encoding="UTF-8"?>
<graph xmlns="http://www.cs.rpi.edu/XGMML" id="1371492552429" label="MirTarBase">
   <att label="Type" name="Type" value="MTIs" type="string" />
   <att label="Database" name="Database" value="miRTarBase 3.5" type="string" />
   <node xmlns="" id="3280" label="3280">
      <att type="list" name="identifiers">
         <att type="string" name="identifiers" value="3280" />
         <att type="string" name="identifiers" value="HES1" />
         <att type="string" name="identifiers" value="Q14469" />
         <att type="string" name="identifiers" value="Q8IXV0" />
         <att type="string" name="identifiers" value="ENSG00000114315" />
      </att>
      <att label="entrezGeneID" name="entrezGeneID" value="3280" type="string" />
      <att label="ensemblID" name="ensemblID" value="ENSG00000114315" type="string" />
      <att label="biologicalType" name="biologicalType" value="gene" type="string" />
      <att label="label" name="label" value="HES1" type="string" />
      <att label="organism" name="organism" value="Homo sapiens" type="string" />
   </node>
   <node xmlns="" id="hsa-miR-449a" label="hsa-miR-449a">
      <att type="list" name="identifiers">
         <att type="string" name="identifiers" value="hsa-miR-449a" />
         <att type="string" name="identifiers" value="hsa-miR-449" />
         <att type="string" name="identifiers" value="MIMAT0001541" />
      </att>
      <att label="biologicalType" name="biologicalType" value="microRNA" type="string" />
      <att label="miRBaseAccession" name="miRBaseAccession" value="MIMAT0001541" type="string" />
      <att label="label" name="label" value="hsa-miR-449a" type="string" />
      <att label="organism" name="organism" value="Homo sapiens" type="string" />
   </node>
   <edge xmlns="" id="3280" label="3280" source="hsa-miR-449a" target="3280">
      <att label="interaction" name="interaction" value="" type="string" />
      <att label="supportType" name="supportType" value="Functional MTI" type="string" />
      <att label="experiments" name="experiments" value="Western blot" type="string" />
      <att label="interactionType" name="interactionType" value="MTI" type="string" />
      <att label="referenceID" name="referenceID" value="21418558" type="string" />
      <att label="miRTarBaseID" name="miRTarBaseID" value="MIRT006346" type="string" />
      <att label="datasource" name="datasource" value="miRTarBase 3.5" type="string" />
   </edge>
</graph>
```
