# Supplementary material for: CyTargetLinker: A Cytoscape App to Integrate Regulatory Interactions in Network Analysis
Source: PLoS One. 2013 Dec 5;8(12):e82160. doi: 10.1371/journal.pone.0082160 (PMC3855388; doi:10.1371/journal.pone.0082160)

Supporting Information: S2

<u>Case study 1</u>: Enrichment of a mouse molecular interaction network, containing genes linked to diabetes mellitus, with miRNA information.

The initial network for case study 1 was obtained using Gene2MeSH (http://gene2mesh.ncibi.org) and the STRING database (http://www.string-db.org).

Genes linked to *Diabetes mellitus* (DM) were obtained using Gene2MeSH. The 23 DM-linked genes were used the input list in the STRING database:

ENSMUSG00000000394, ENSMUSG00000022136, ENSMUSG00000032872, ENSMUSG00000033685, ENSMUSG00000041798, ENSMUSG00000047591, ENSMUSG00000029644, ENSMUSG00000027596, ENSMUSG00000037852, ENSMUSG00000012705, ENSMUSG00000044167, ENSMUSG00000018566, ENSMUSG00000028645, ENSMUSG00000027690, ENSMUSG00000005534, ENSMUSG00000038894, ENSMUSG00000022878, ENSMUSG00000028556, ENSMUSG00000035804, ENSMUSG00000000215, ENSMUSG00000022383, ENSMUSG00000059201, ENSMUSG00000057722

STRING (version 9) contains interactions between 18 out of 23 genes (see network below):

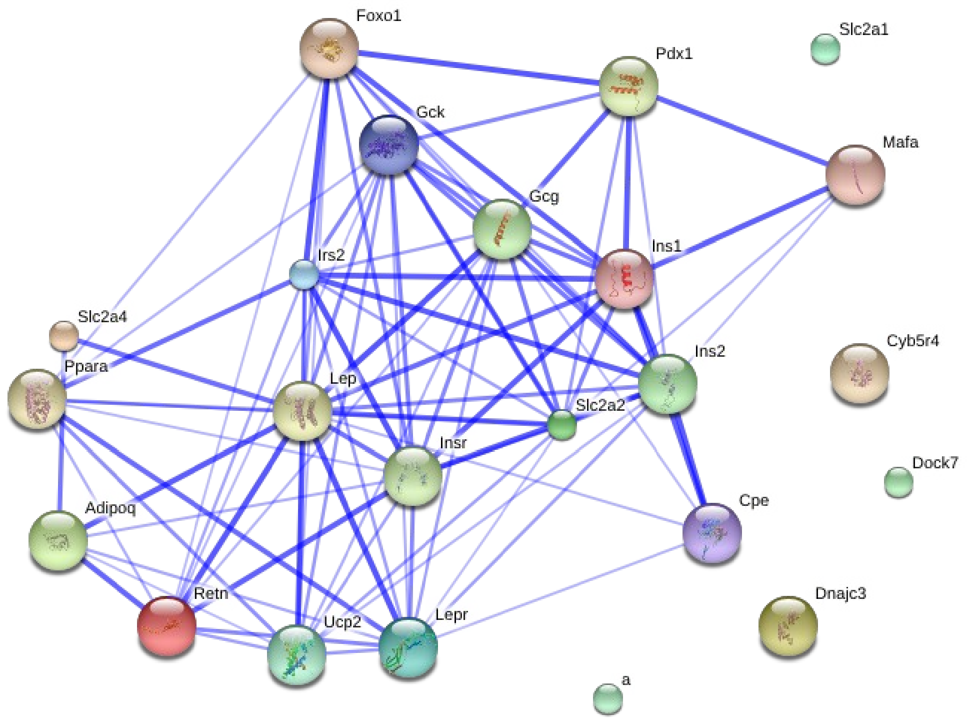

This network was downloaded and opened in Cytoscape. The network was extended with microRNA-target interactions.

CyTargetLinker added 700 new interactions from

- microCosm (486) *predicted interactions*
- TargetScan (182) *predicted interactions*
- miRTarBase (5) *validated interactions*
- miRecords (2) *validated interactions*

Figure 2 in the manuscript shows the threshold overlap which contains only interactions that are supported by at least two of the databases. The VENN diagram below shows how many interactions are present in more than one database. It shows that the overlap between the databases is very low, because most of the interactions are only present in one specific database. That also indicates that by using one specific database users might lose interactions that could be of interest.

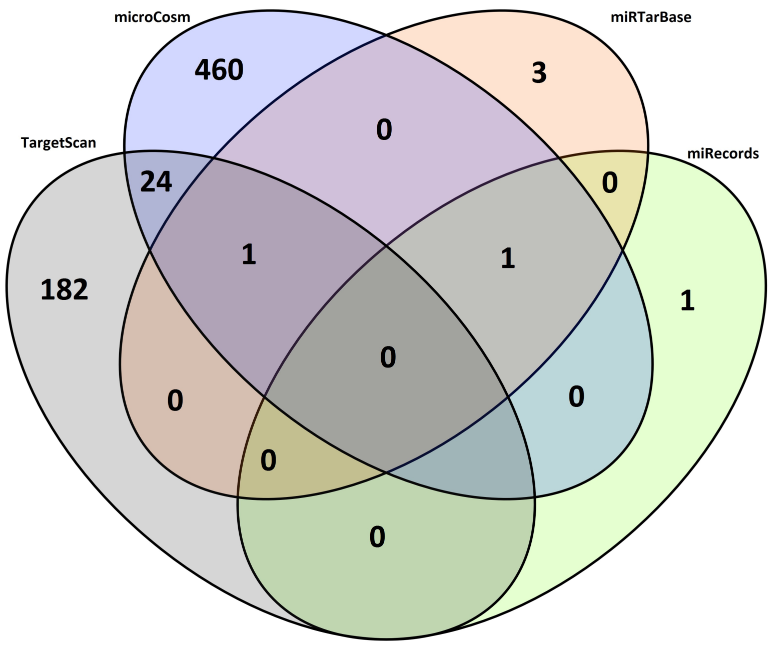

Supplement: File S2 — Supporting information for case study 1 which is described in the results section. Description of the initial network of the use case and the overlap between the different miRNA-target databases for this use case. (PDF) [file pone.0082160.s002.pdf]
