# Supplementary material for: CyTargetLinker: A Cytoscape App to Integrate Regulatory Interactions in Network Analysis
Source: PLoS One. 2013 Dec 5;8(12):e82160. doi: 10.1371/journal.pone.0082160 (PMC3855388; doi:10.1371/journal.pone.0082160)
Supplement: File S3 — Supporting information for case study 2 which is described in the results section. Description of the initial network of the use case and more detailed information about the transcription factors, e.g. transcription factor families. (PDF) [file pone.0082160.s003.pdf]

Supporting Information: S3

Case study 2: Extension with ENCODE TF regulation information of a molecular interaction network of human DNA repair genes and its analysis.

In this example, we used a set of DNA repair genes as input for the GeneMANIA app in Cytoscape. The query genes are:

NBN, MRE11A, H2AFX, DMC1, RAD54B, RAD50, MSH2, PMS2, MSH6, MSH3, PCNA, BRCA2, FEN1, BARD1, RAD51, RAD54L, XRCC2, RAD51C, XRCC3, RAD51AP1

The initial network is depicted below:

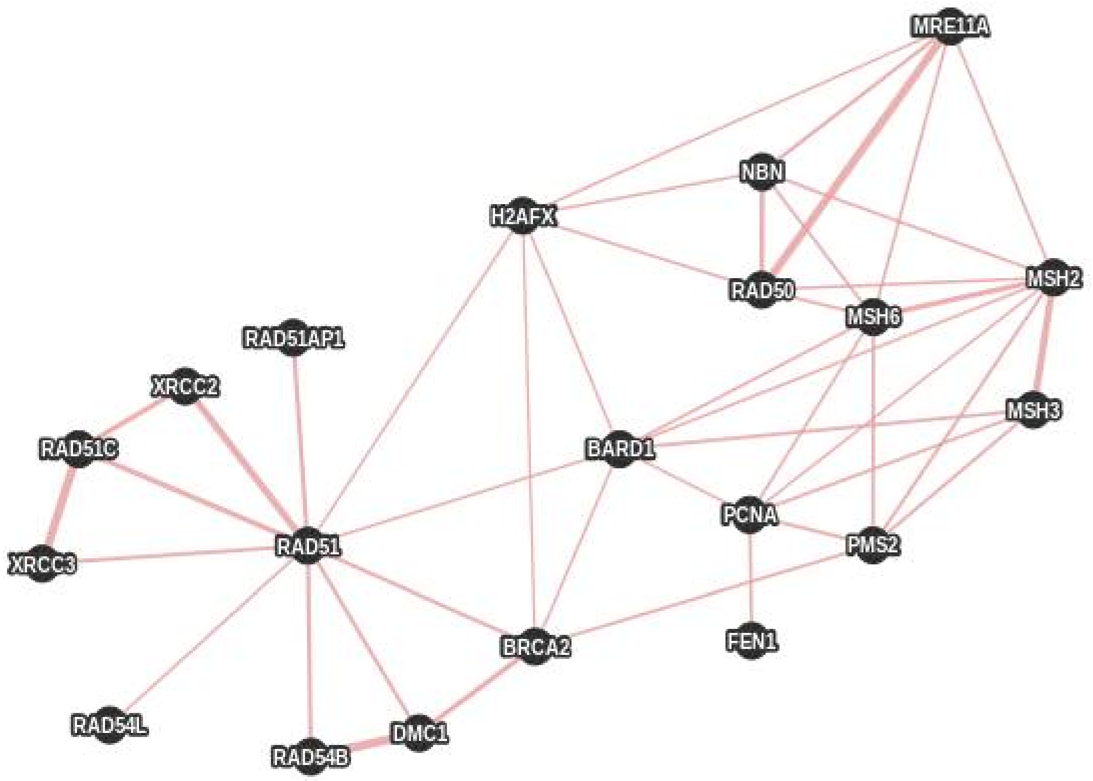

The network was extended with proximal and distal transcription factor regulators from ENCODE. The regulatory interaction data was published by Gerstein *et al* in 2012 in Nature ("Architecture of the human regulatory network derived from ENCODE data", http://dx.doi.org/10.1038/nature11245).

35 transcription factors were added that are regulating the query genes proximal or distal, see Figure 3 in the manuscript.

In this use case there are only 4 TF that regulate genes in a distal way:

EP300 → MSH2
CEBPB → MSH2
CTCF → NBN
RAD21→ NBN

We also looked at the transcription factor family categorization obtained from ENCODE. The three top families in the extensions are:

- *7 transcription factors with a zink finger structure*
- *5 transcription factors with a helix loop helix structure*
- *4 transcription factors with a bZIP domain*
