# Supplementary material for: CyTargetLinker: A Cytoscape App to Integrate Regulatory Interactions in Network Analysis
Source: PLoS One. 2013 Dec 5;8(12):e82160. doi: 10.1371/journal.pone.0082160 (PMC3855388; doi:10.1371/journal.pone.0082160)

Supporting Information: S4

<u>Case study 3</u>: Enrichment of a human pathway from WikiPathways with miRNAs, TFs and drugs targeting the genes and gene products in the pathway

---

In use case 3 we extended the ErbB signaling pathway from WikiPathways (http://www.wikipathways.org) with microRNA-target, transcription factor-target and drug-target interactions.

CyTargetLinker created a meta-network containing the following interactions

- 138 drug-target
- 158 transcription factor-target
- 162 validated microRNA-target

The pathway was converted to a Cytoscape network with the GPML plugin for Cytoscape 2.8. In the new Cytoscape 3 version this plugin was renamed as *WikiPathways app* and was added to the Cytoscape app store recently.

The pathway diagram below shows the original ErbB Signaling Pathway on WikiPathways (http://www.wikipathways.org/instance/WP673_r69914).

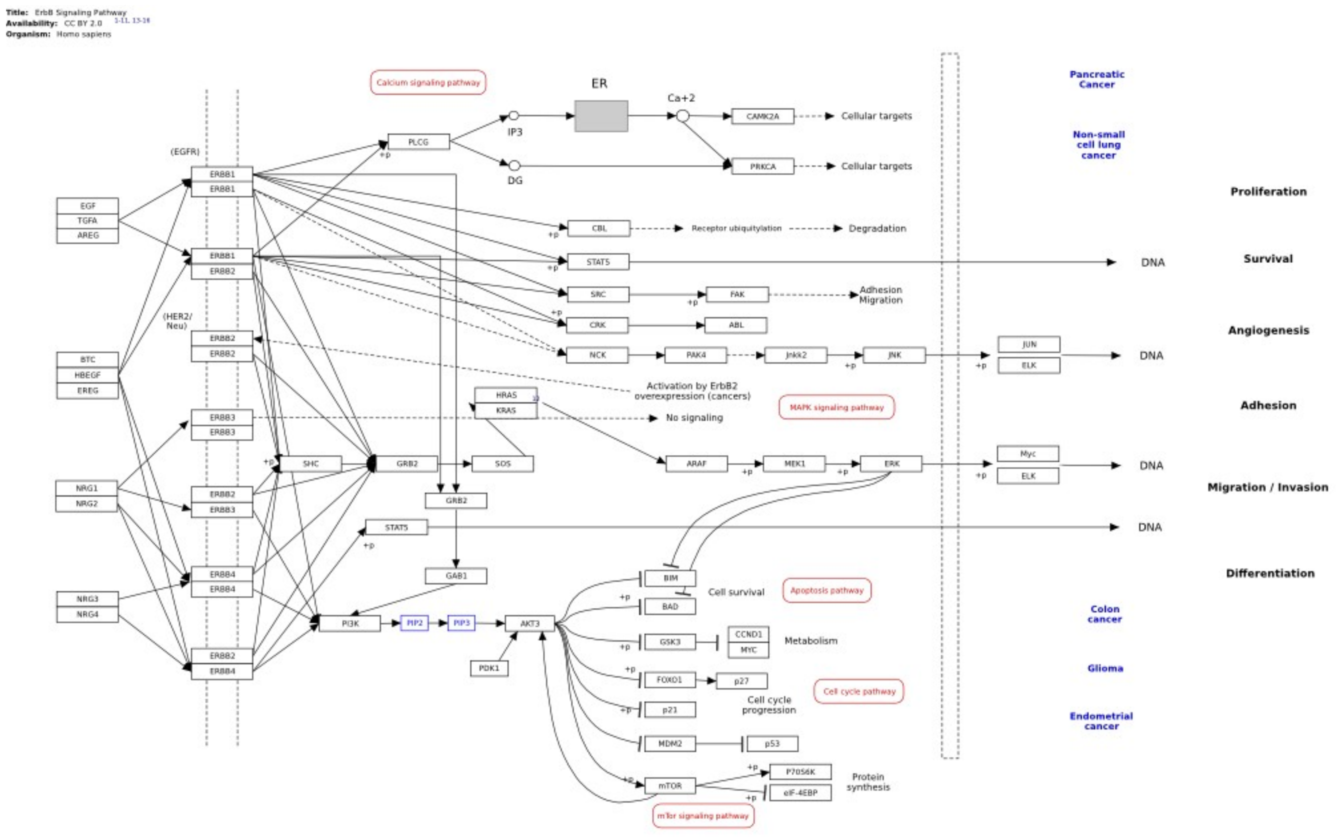

Supplement: File S4 — Supporting information for case study 3 which is described in the results section. Contains the pathway diagram of the ErbB signaling pathway from WikiPathways. (PDF) [file pone.0082160.s004.pdf]
